# Nutrition Focused Physical Examination Components Specific to Older Adults: A Modified Delphi Study

**DOI:** 10.1111/jhn.70101

**Published:** 2025-07-22

**Authors:** Christina E. Gassmann, Caroline M. Kiss, Alainn Bailey, Laura Byham‐Gray, Diane L. Rigassio Radler

**Affiliations:** ^1^ Department of Nursing and Allied HealthCare Professions University Hospital Zurich Zurich Switzerland; ^2^ University Department of Geriatric Medicine FELIX PLATTER Basel Switzerland; ^3^ Department of Clinical and Preventive Nutrition Sciences, School of Health Professions Rutgers University Newark New Jersey USA

**Keywords:** consensus, gerontology nutrition, healthy aging, nutrition assessment, older adults, physical signs

## Abstract

**Background:**

The number of older adults will double worldwide by 2050, impacting the social system with higher healthcare personnel needs and economic costs. Registered dietitian nutritionists play a critical role in the holistic management of chronic diseases and related nutritional problems. Thereby, a comprehensive nutritional assessment should include a nutrition‐focused physical examination (NFPE) to ensure early identification of nutrition‐related problems. However, explicit NFPE components across different assessment categories (NFPE+) of the Nutrition Care Process for older adults are lacking.

**Methods:**

This modified Delphi study aimed to identify NFPE+ components specific to older adults (≥ 65 years) through a literature review and to obtain consensus about the relevance and usefulness of these components among international dietitian experts in gerontology nutrition. Experts rated how useful and relevant the identified NFPE+ domains and components were using a 5‐point Likert‐type scale. Open‐ended answers allowed the experts to suggest new components or propose adjustments to the identified components. Consensus was defined a priori at 75% agreement.

**Results:**

The literature review identified 30 NFPE+ components specific to older adults. The Delphi panel comprised of 15 experts from eight countries with 23.5 (±11.5) years of experience in geriatric nutrition. All 30 proposed NFPE+ components and three newly suggested components achieved consensus through two Delphi rounds. These 33 components were categorized into five NFPE+ domains in a finalized NFPE+ tool.

**Conclusions:**

NFPE+ domains and components specific to older adults may significantly contribute to a focused and comprehensive nutritional assessment in older adults.

## Summary

- Thirty‐three useful and relevant NFPE+ components categorized into five NFPE+ domains (*General*, *Functional Status/Body Composition*, *Dehydration*, *Edema*, and *Oral Health*) specific to older adults met consensus by fifteen experts in geriatric nutrition care from across the globe through a modified Delphi survey.
- NFPE+ domains and components specific to older adults may significantly contribute to a comprehensive nutritional assessment in older adults.

## 1 Introduction

By the year 2050, it is estimated that the number of adults aged 60 years and older will double worldwide, and the number of people over 80 years will triple [1]. Additionally, many older adults suffer from multiple chronic diseases (defined as ≥ 2 chronic conditions) [2]. For example, in the United States of America, the proportion of older adults with multiple chronic diseases is expected to increase by more than 90% from 7.8 million in 2020 to almost 15 million in 2050 [3]. This increase in older adults, with or without chronic diseases, impacts the social system with higher healthcare personnel needs, social burden, and economic costs [4, 5, 6]. Older adults with chronic diseases are particularly affected by nutritional problems such as decreased dietary and fluid intake, poor appetite, altered nutrient requirements, and age‐induced body composition alterations, leading to malnutrition, dehydration, micronutrient deficiencies, and sarcopenia [6, 7, 8, 9, 10]. Compared to younger adults, in older adults, nutrition‐related problems impact overall health more commonly, functional status declines more rapidly, and as a result, recovery from nutrition‐related problems or acute illnesses is prolonged [6, 9]. The older the individuals are, and the more chronic diseases are present, the more difficult recovery gets [6, 11]. In this context, some publications classify older adults into “young‐old” (65–74 years), “old‐old” (75–84 years), or “oldest‐old” (> 84 years) [12, 13], to highlight that “young‐old” individuals may be different than the “oldest‐old”.

Registered dietitian nutritionists (RDN) or nutrition professionals with equivalent degrees (e.g., “Accredited Practising Dietitian” in Australia, or “Registered Dietitians” in the United Kingdom) play a critical role in the holistic management of chronic diseases and related nutritional problems to achieve positive clinical outcomes, enhance quality of life (QoL), and reduce costs [5, 6, 7, 14, 15]. A comprehensive nutritional assessment is an integral part of the Nutrition Care Process (NCP) and includes a physical examination to ensure early and proper identification of nutrition‐related problems [16, 17, 18]. While the American Standards of Practice and Standards of Professional Performance for RDN in Post‐Acute and Long‐Term Care Nutrition include indicators for a nutrition focused physical examination (NFPE) [14], the listed components for older adults are identical to those for younger adults [5, 6, 14, 19, 20, 21, 22]. Additionally, distinct criteria for categorizing findings into normal or abnormal, specific to older adults, are lacking [5, 19, 20, 21]. Distinct NFPE components would help to differentiate between healthy aging individuals and those who need further assessment. For example, standard physical tests, typically used in adults to assess dehydration, might not be as sensitive and reliable in older adults [6, 9, 23]. The European Society for Clinical Nutrition and Metabolism (ESPEN) guideline recommends against traditional physical tests and signs [9]. Instead, ESPEN highlights direct serum/plasma osmolality measurement as the gold standard for dehydration detection [9]. However, other publications recommend using a combination of clinical signs to screen for dehydration in older adults, particularly in the absence of laboratory test availability [6, 23]. These physical signs and tests are vital for assessing dehydration in nursing home residents or other settings where relevant diagnostic methods are not feasible [6].

Besides hydration, maintaining functional status is also a top priority in this population to ensure healthy aging and QoL [9, 24, 25]. Body composition changes with age, particularly with advanced age [26, 27, 28, 29, 30, 31]. Muscle mass decreases, and fat mass increases [26, 27, 32, 33], although this age‐related change in body composition is less pronounced in the robust elderly than in individuals with chronic diseases [26]. Normal age‐related change in body composition is associated with a lower risk of chronic diseases, impaired functional status, and mobility [5, 26, 34, 35, 36, 37, 38, 39, 40, 41]. Compared to younger adults, nutrition‐related problems in older adults impact their overall health status more quickly and to a greater extent due to a reduced compensation capacity in old age [6, 9]. Nutritional problems or acute or chronic illness can lead to a downward spiral in functional status [6, 9]. Particularly with increasing age, recovery can take significantly longer or might be impossible [6, 9]. With newer guidelines for sarcopenia, not only muscle mass but also muscle strength and functional status assessment are recommended in older adults [5, 16, 42].

Physical exam findings, which comprise the NFPE, are an integral part of the NCP. However, relevant and useful nutritional assessment of physical components in older adults go beyond the traditional head‐to‐toe NFPE components. This study aimed not to replace the traditional head‐to‐toe NFPE components for adults but to determine additional NFPE (NFPE+) across the NCP assessment categories, complementing the holistic nutritional assessment in this population.

Due to the limited evidence for explicit NFPE+ components in older adults, the Delphi technique was chosen as this method is widely used and known for delivering valuable results in health care [43, 44]. Therefore, this modified Delphi study aimed to, first, identify NFPE+ components specific to older adults (≥ 65 years) through a practice‐informed literature review and, second, to use the Delphi technique to obtain consensus about the relevance and usefulness of these components among international experts in gerontology nutrition.

## 2 Methods

### 2.1 Literature Review and Delphi Preparation

To identify an initial list of NFPE+ domains and components, a practice‐informed literature review was conducted in PubMed, Cumulative Index to Nursing and Health Literature (CINAHL), and Cochrane databases. The databases were searched with a combination of Medical Subject Headings (MeSH) terms and keywords, including terms related to NFPE+ components specific to older adults, such as ‘Functional status’, ‘Body Composition’ [MeSH], ‘Oral Health’, ‘Dehydration’, ‘Physical examination’ [MeSH], or ‘Older adults’.

In health care, the Delphi technique is a scientific research method aimed at managing an organized expert panel communication process to achieve consensus on clinical practice recommendations [43, 44] metacarpal fracture The Delphi technique is widely used and known for being a relevant method to achieve valuable results, especially in situations with limited evidence [43, 44, 45]. While the Delphi statements in the conventional Delphi technique are expert‐based, the Delphi statements in the modified Delphi technique used in this study were identified through a comprehensive, practice‐informed literature search [43]. The Delphi method is often conducted with 10–30 experts for best practice questions in healthcare [43, 44, 46]. To account for potential drop‐outs, the aim was to find 15 to 30 qualified and representative experts to participate in the Delphi survey rounds. Experts were sought internationally based on defined inclusion criteria: Experts had to be RDNs (or equivalent degrees) with at least 5 years of clinical experience in the nutritional care of geriatric patients. Exclusion criteria were if experts were not confident in the nutritional assessment of physical components.

Experts were identified through purposeful sampling, personally invited, and given 3 weeks to consider their participation. One recruitment route was through dietetic practice groups, such as members of the American Academy of Nutrition and Dietetics «Dietetics in Health Care Communities» practice group, the “Older People Specialist Group” by the British Dietetic Association and Committee members of the European Sustainable Development Network “Older Adults”. Additionally, experts were identified through relevant literature/guidelines in the field. The authors of this publication were not included in the expert panel.

### 2.2 Ethics and Consenting Language

The Institutional Review Board (IRB) of Rutgers University, New Jersey, United States of America, approved the study protocol, including the expert recruitment process (Pro2024000274). The entire study was conducted following the Declaration of Helsinki, whereby all data were kept confidential, anonymized, and protected. The first electronic internet‐based Delphi survey round included the letter of consent followed by a question asking the participants if they agreed to participate.

### 2.3 Survey Instrument

Based on the identified and grouped NFPE+ components and domains, a questionnaire was generated as an online survey in Qualtrics (Qualtrics Survey Software, Provo, Utah) [47], an online survey tool. After creating the survey, a pre‐test was conducted with Rutgers faculty experts with long‐standing research experience, particularly regarding surveys and NFPE.

For the first Delphi round, the survey comprised questions about sociodemographic and professional characteristics. The remainder of the survey focused on gaining consensus for the NFPE+ domains and components by presenting the pre‐identified NFPE+ components grouped into the four domains *General, Functional Status/Body Composition, Dehydration*, and *Oral Health*. Brief information on conducting the assessment and potential indicators or cutoff values for abnormal findings were described. The panelists were asked to anonymously rank the usefulness and relevance of each identified domain and component quantitatively using a 5‐point Likert scale ranging from ‘strongly disagree’ (1), ‘somewhat disagree’ (2), ‘neither disagree nor agree’ (3), ‘somewhat agree’ (4), to ‘strongly agree’ (5). In clinical practice with different practice settings (acute care, ambulatory/outpatient care, long‐term care, etc.), nutritional assessment indicators must be both useful (possible to be applied in clinical practice) [48], as well as relevant (appropriate and significant in clinical practice) [49]. Therefore, the experts were asked to rank each component and each domain both for its usefulness and relevance. For each domain and each component, one question on the usefulness and one on the relevance were included. Additionally, instructions for the experts were provided at the beginning of the survey and throughout the survey.

Open‐ended questions allowed the experts to explain their rating qualitatively, suggest new components, or recommend components to be excluded. To capture the experts' opinion on the difference between ‘young‐old’, ‘old‐old’, and ‘oldest‐old’ individuals [12, 13], the experts were also asked whether all the identified domains and components were useful and relevant for all three age categories. All questions in the online survey were designed with no forced responses.

Components that reached a priori‐defined consensus (consensus determination is described under «Statistics») were retained and were not presented again in the subsequent rounds. All other components and the identified themes from the open‐ended question were presented again in the following survey round for re‐evaluation by all experts. The authors planned for up to three rounds of the Delphi process if consensus was not achieved with rounds one and two.

### 2.4 Survey Process

**Figure 1:**
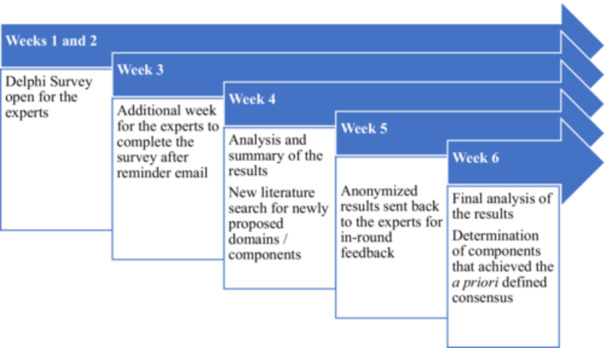
Delphi survey process.

The survey was iterative and involved two rounds of questioning, each lasting 6 weeks (Figure 1). The second round started directly after the first round ended. The content and components for the second Delphi round were defined by summarizing the group responses from the first round [44]. The invited experts were sent an email with an individual link to the survey and asked to complete the survey within 3 weeks, with weekly reminder emails. All experts who had not participated in the survey after this 3‐week limit were excluded from further participation.

After each Delphi round, the results were statistically summarized. If additional components or suggestions for adaptation were proposed, a new literature search was carried out to evaluate each proposed component for inclusion. All domains, components, and the identified themes from the open‐ended question were returned to the experts anonymously for peer feedback. The experts could see their own ratings along with the overall deidentified average results. The experts then had another week to reconsider their ratings or add further comments. At the end of this week, it was determined whether individual components had already reached the a priori defined consensus. Those components that achieved the a priori consensus were maintained and not presented or visible again for rating and commentary in the second round.

The second survey round again contained NFPE+ components and domains to be rated, and open‐ended questions allowed for suggestions for revisions or other open comments. An overview of the domains and components that had already reached consensus in the first round was included in the survey. At the end of the second round, all results were again returned to the experts anonymously, whereby the experts had another week to reconsider their ratings or add further comments.

### 2.5 Statistics and Consensus Definition

The results were statistically summarized using IBM SPSS Statistics for Windows, Version 29.0 [50]. The sociodemographic and professional data were analyzed using descriptive statistics. Continuous variables were reported as mean, standard deviation, median, interquartile range (IQR), minimum, and maximum. Categorical variables were reported as total count (N) and percentage (%). Statistical analysis of the usefulness and relevance ranking included each response's frequency, percentage of agreement, mean, and standard deviation. Open‐ended questions were analyzed and presented in the following rounds using content analysis.

Consensus was defined a priori at 75% agreement among the panelists, as this cutoff has been used in previous publications [44, 51, 52]. This required that for each component or domain, at least 75% of the panelists responded ‘neither disagree nor agree’, ‘somewhat agree’, or ‘strongly agree’ on the 5‐point Likert‐type scale or that the component ranked within ± 1 SD of ‘somewhat agree’, or ‘strongly agree’.

## 3 Results

### 3.1 Expert Panel

The invitation to participate in the Delphi study was sent to two dietetic practice groups and as a personal invitation to 20 experts. Of the 20 personally invited experts, 12 responded, with all experts meeting the inclusion criteria. From within the two dietetic practice groups, 19 responded, but 10 had to be excluded (seven due to incomplete answers to the inclusion criteria and three due to less than 5 years of experience in geriatric nutrition). Of the 21 experts included, 15 participated in the first Delphi round (Table 1) and 11 in the second Delphi round. Panelists came from eight different countries, with five (33.4%) panelists from the United States of America, three (30%) from the United Kingdom, two (13.3%) from Switzerland, and one panelist each (6.7%) from Australia, Israel, Turkey, Poland, and Portugal. The expert panel had extensive years (23.5 ± 11.5 years) of experience in geriatric nutrition.

**Table 1:** Sociodemographic and professional characteristics of the expert panelists of the first Delphi round (*N* = 15).

Variable		*n* (%)
Gender^a^		
Female		15 (100.0)
Total		15 (100.0)
Current continent of employment		
Europe and Asia		9 (60.0)
America and Australia		6 (40.0)
Total		15 (100.0)
Highest degree earned		
Bachelor		6 (40.0)
Master		3 (20.0)
Doctorate/PhD		6 (40.0)
Total		15 (100.0)
Primary practice setting		
College/University teaching		4 (26.7)
Long‐term care or extended care, or assisted living facility		3 (20.0)
Other^b^		3 (20.0)
Acute/inpatient care		2 (13.3)
Food Service Management		1 (6.7)
School Nutrition		1 (6.7)
Community or public health program		1 (6.7)
Total		15 (100.0)
Advanced and specialist credentials		
No		12 (80.0)
Yes^c^		3 (20.0)
Total		15 (100.0)
Confidence in assessing nutrition‐related physical components in older adults^d^		
Extremely confident		8 (53.3)
Somewhat confident		7 (46.7)
Total		15 (100.0)
Frequency of assessing nutrition‐related physical components in older adults		
> 20 times monthly		3 (20.0)
11–20 times monthly		1 (6.7)
6–10 times monthly		4 (26.7)
< 5 times monthly		4 (26.7)
Never		3 (20.0)
Total		15 (100.0)
Variable	Mean ± SD	Median (IQR)
Age (years)	46.1 ± 12.9	50.0 (34.0–54.0)
Years as an RDN or equivalent degree (years)	23.5 ± 13.0	26.0 (11.0–31.0)
Years in geriatric nutrition practice (years)	23.5 ± 11.5	23.0 (15.0–33.0)

*Note:* Percentages may not add up to 100% due to rounding.

Abbreviations: IQR, interquartile range; PhD, Doctor of Philosophy; RDN, Registered Dietitian Nutritionist; SD, standard deviation

^a^ No participants were male, transgender, nonbinary/third gender, or preferred not to answer.

^b^ 2: More than one setting, 1: Post Acute.

^c^ 2: Certified Specialist in Gerontological Nutrition (CSG), 1: Specialized in Nutrition‐Focused Physical Examination for Malnutrition.

^d^ No participants were unconfident since this was an inclusion criterion for expert panellists.

### 3.2 Identified NFPE+ Specific to Older Adults

The practice‐informed literature review identified 30 NFPE+ components specific to older adults, which were grouped into four domains: *General*, *Functional Status/Body Composition*, *Dehydration*, and *Oral Health*. Table 2 provides an overview of the four domains, including the identified NFPE+ components, potential indicators or cutoff values for abnormal findings, and brief important information on conducting the assessment.

**Table 2:** Identified NFPE+ domains and components specific to older adults (≥ 65 years).

Domain	Component	Abnormal Signs/Symptoms	Assessment Notes
General			
	Overall medical condition impacting health, independence, and nutrition status [7, 14]:	No cutoff, range from 1 (very fit) to 9 (terminally ill), with each level representing a different degree of frailty [53]	
	‒ Clinical frailty scale [53]		
Functional status/Body composition			
Capacity in own social and physical context	ADLs [14, 54]		Examples of tools:
			‒ Barthel Index [55] ‒ Katz Index of Independence in Activities of Daily Living [56]
	IADLs [54]		Example of tool: The Lawton IADL scale [57]
Physical performance and muscle strength	Handgrip [5, 7, 14, 15, 16, 20, 24, 58, 59, 60]	Use sex‐, age‐, and ethics‐specific cutoffs and references [5] Cutoffs for sarcopenia with Jamar dynamometer: < 27 kg for males [5, 9] < 16 kg for women [5, 9] < 28 kg for Asian males [7, 10] < 18 kg for Asian women [7, 10]	Might need to be adjusted to BMI (especially in suspected sarcopenic obesity) [61] Most suitable instrument (Pneumatic, Hydraulic, Spring Type, Strain Gauge) for older adults needs further research as an alternative to the classic Jamar dynamometer [62, 63]
	Chair stand test [16, 24]	> 15 s for five rises [5, 60] ≥ 12 s for five rises [16, 24]	
	Balance test [5]: 4‐stage balance test [64]	> 10 s for each of the four positions [64]	No assistive device should be used, and eyes should be kept open during the test [64]
	400 m walk test [5, 60]	≥ 6 min [5, 60]	
	SPPB [5, 16, 60]	≤ 8 points [5, 60] ≤ 9 points [16]	
	TUG [5]	≥ 20 s [5]	Walking aids allowed
	Gait speed [8, 10]: 4 m walking test [10, 13, 60] 6 m walking test [16, 24]	≤ 0.8 m/s [5, 60] < 1.0 m/s [16, 24]	
Image methods	Muscle mass [5, 16]:	Appendicular skeletal muscle mass [5, 16]:	
	‒ Dual‐energy X‐ray ‒ Absorptiometry [5, 16] ‒ Computed tomography [5, 16] ‒ Magnetic resonance imaging [5, 16] ‒ Bioelectrical impedance analysis [5, 16] ‒ Ultrasound [5, 16]	‒ Men < 7.0 kg/m^2^ (when measured with Dual‐energy X‐ray absorptiometry or with bioelectrical impedance analysis) [16] ‒ Women < 5.4 kg/m^2^ (when measured with Dual‐energy X‐ray absorptiometry) [16] or < 5.7 kg/m^2^ (when measured with bioelectrical impedance analysis) [16] ‒ Men: < 20 kg or < 7.0 kg/m^2^ [5] ‒ Women: < 15 kg or < 5.5 kg/m^2^ [5]	
Anthropometrics			Anthropometrics in general less sensitive, but more suitable/applicable in many (clinical) settings than image methods [20] Use sex‐, age‐, and ethics‐specific cutoffs and references [20] Muscle circumferences might be difficult to assess in patients with obesity or edema [20, 24]
	BMI [14]	< 22 (kg/m^2^) [9] < 23 (kg/m^2^) [65]	
	MUAC [14, 20]		
	Calf circumference [5, 16, 20, 24, 60]	< 33 cm in males [20] < 32 cm in females [20] < 34 cm in males [16, 24, 60] < 33 cm in females [16, 24, 60]	In adults with overweight or obesity, decrease the measured value by 3 cm (BMI, 25–30) or 7 cm (BMI, 30–40) [20, 66]
	Waist circumference [61]	Disease risk for type 2 diabetes, hypertension, and CVD [61, 67]: Men ≤ 102 cm [61, 67] Women ≤ 88 cm [61, 67] Two levels for Asians [61]: I. (Increased relative risk for development of obesity‐associated risk factors) ≥ 78 cm for Males ≥ 72 cm for Females II. (Cutoff points associated with ≥ 1 CVD risk factor present) ≥ 90 cm for Males ≥ 80 cm for Females	Abnormal Signs/Symptoms not specific to older adults but to all adults [61] Screen in combination with sarcopenia indicators/tools to evaluate risk of sarcopenic obesity [61]
Dehydration			
	Vital signs [6, 23]	Higher pulse (> 90 beats/min.) [6, 23] Increased respiratory rate (> 20 breaths/min) [6, 23] Decreased blood pressure (< 100 mmHg) [6, 23]	Use a combination of clinical findings [6] Step‐by‐step diagnostic strategy: Combine anamnestic items and physical symptoms and confirm findings with blood tests to diagnose dehydration [6] (Measured serum or plasma osmolality [9, 21] or calculated serum or plasma osmolarity [9, 21]) Use NFPE+ mainly when laboratory tests are not possible [6], or use laboratory tests to confirm physical findings
	Rapid weight change [6]	Weight loss > 1 kg/d [6]	
	Nails: Capillary refill [23]	Refill time ≥ 2 s [23]	
	Oral mucosa [6]	Dry mucosa (not caused by meds) [6]	
	Tongue [6]	Dry longitudinal furrowed tongue (not caused by meds) [6]	
	Temperature peripheries [23]	Cold peripheries [23]	
	Incontinence pad/urine output [6]	Dry incontinence pad/decreased urine output [6]	
	Urine color [6]	Change in urine color [6]	
	Behavior [6]	Change in behavior (e.g., more confused and/or consciousness) [6]	
Oral Health			
	Screening/Assessment tools [17]	Revised Oral Assessment Guide (ROAG) or Oral Health Assessment Tool (OHAT) [17]	
	Taste and smell [17, 68]	Taste alterations [68]	

Abbreviations: ADL, activities of daily living; BMI, body mass index; Cm, centimeter; CVD, cardiovascular disease; D, day; IADL, instrumental activities of daily living; Kg, kilogram, M, meter; Min, minute; MmHg, millimetres of mercury; MUAC, mid‐upper arm circumference; NFPE, nutrition focused physical exam; S, second; SPPB, short physical performance battery; TUG, timed up & go.

### 3.3 Delphi Rounds

The first Delphi round analysis showed that all panelists had completed all rating questions on the 5‐point Likert scale. Consensus was reached in the first round for all four proposed domains (*General, Functional Status/Body Composition*, *Dehydration, and Oral Health*) and 24 of the 30 proposed components (Table 3). All six components that did not reach consensus were from the domain *Functional Status/Body Composition* in the subdomain *Muscle Mass/Body Composition* and included the components *dual‐energy X‐ray absorptiometry (DEXA)*, *computed tomography (CT), magnetic resonance imaging (MRI), bio‐electrical impedance analysis (BIA), ultrasound*, and *waist circumference*.

**Table 3:** NFPE+ components and domains specific to older adults—consensus determination after rounds 1 and 2 (round 1 *N* = 15, round 2 *N* = 11).

Domain & component of examination	Usefulness^a^ mean	Standard deviation	Frequency (%) rating ≥ 3	Relevance^b^ mean	Standard deviation	Frequency (%) rating ≥ 3	Consensus
**Domain 1 general**	4.73	0.59	100.0% (15/15)	4.67	0.62	100.0% (15/15)	Yes
**Components domain 1 general**							
1 Clinical frailty scale	4.33	0.90	93.3% (14/15)	4.20	0.86	93.3% (14/15)	Yes
**Exemplary panelist quotes extracted verbatim**							
*General health and presence of a frailty diagnosis are very relevant and impact nutritional status of older adults*.							
**Expert panelist feedback: no suggested revisions**							
**Domain 2 functional status/body composition**	4.33	0.82	93.3% (14/15)	4.27	0.88	93.3% (14/15)	Yes
**Components domain 2 functional status/body composition**							
**Subdomain capacity in own social and physical context**	4.33	1.05	93.3% (14/15)	4.33	1.05	93.3% (14/15)	Yes
1 Activities of daily living	4.53	0.83	93.3% (14/15)	4.4	0.91	93.3% (14/15)	Yes
2 Instrumental activities of daily living	4.40	0.83	93.3% (14/15)	4.40	0.83	93.3% (14/15)	Yes
**Subdomain physical performance and muscle strength**	4.27	0.88	93.3% (14/15)	4.27	0.88	93.3% (14/15)	Yes
3 Handgrip strength	4.27	0.70	100.0% (15/15)	4.13	0.64	100.0% (15/15)	Yes
4 Chair stand test	4.00	0.93	86.7% (13/15)	3.73	0.88	86.7% (13/15)	Yes
5 Balance test	3.40	0.91	86.7% (13/15)	3.27	0.88	86.7% (13/15)	Yes
6 Walk test (4–400 m)	3.53	0.64	93.3% (14/15)	3.40	0.63	93.3% (14/15)	Yes
7 Short physical performance battery test	3.73	0.80	93.3% (14/15)	3.53	0.74	93.3% (14/15)	Yes
8 Time up and go test	3.80	0.68	93.3% (14/15)	3.80	0.76	93.3% (14/15)	Yes
9 Gait speed	3.60	0.99	80.0% (12/15)	3.40	0.83	80.0% (12/15)	Yes
**Subdomain Muscle mass/Body Composition** *	4.64 (*N* = 11)	0.51	100% (11/11)	4.45 (*N* = 11)	0.93	100% (11/11)	Yes*
10 Dual‐energy X‐ray absorptiometry*	4.00 (*N* = 11)	0.89	90.9% (10/11)	4.20 (*N* = 10)	0.63	100% (10/10)	Yes*
11 Computed tomography*	3.45 (*N* = 11)	1.04	81.8% (9/11)	3.80 (*N* = 10)	1.03	90% (9/10)	Yes*
12 Magnetic resonance imaging*	3.55 (*N* = 11)	1.21	72.7% (8/11)	3.70 (*N* = 10)	1.16	80% (8/10)	Yes*
13 Bio‐electrical impedance analysis*	3.64 (*N* = 11)	1.29	81.8% (9/11)	3.40 (*N* = 10)	1.17	80% (8/10)	Yes*
14 Ultrasound*	3.36 (*N* = 11)	0.67	90.9% (10/11)	3.10 (*N* = 10)	0.74	80% (8/10)	Yes*
15 Body mass index	3.53	1.19	80.0% (12/15)	3.20	1.37	66.7% (10/15)	Yes
16 Mid‐upper arm circumference	3.93	1.03	86.7% (13/15)	3.93	0.88	93.3% (14/15)	Yes
17 Calf circumference	3.93	0.88	93.3% (14/15)	3.93	0.88	93.3% (14/15)	Yes
18 Waist circumference*	3.27 (*N* = 11)	1.01	81.8% (9/11)	3.30 (*N* = 10)	0.95	80% (8/10)	Yes*
**Exemplary panelist quotes extracted verbatim**							
*The functional status of an older person strongly influences their ability to provide, prepare and consume food and should therefore be assessed*.							
*Absolutely required for nutritional assessment and malnutrition evaluation for elderly populations*.							
*Many of these test types are not readily available to request due to cost*.							
*Image methods may not be feasible in some contexts for the assessment of muscle mass in older adults*.							
*If images are available anyways for a patient, I might use them as part of the nutritional assessment. But I wouldn't take those images (or ask for them to be taken) only for the use as part of nutritional assessment due to cost and radiation (DEXA/CT). Plus it might be more time‐consuming to evaluate those images or require a special program to evaluate them compared to easier and faster tests like arm/calf circumference*.							
**Expert panelist feedback: no suggested revisions**							
**Domain 3 dehydration**	4.73	0.59	100.0% (15/15)	4.80	0.56	100.0% (15/15)	Yes
**Components domain 3 dehydration**							
1 Vital signs	3.80	1.27	86.7% (13/15)	3.80	1.27	86.7% (13/15)	Yes
2 Rapid weight loss	4.40	0.99	93.3% (14/15)	4.33	0.98	93.3% (14/15)	Yes
3 Capillary refill	3.53	1.06	86.7% (13/15)	3.47	0.92	86.7% (13/15)	Yes
4 Dry oral mucosa	3.80	1.21	86.7% (13/15)	3.80	1.08	93.3% (14/15)	Yes
5 Longitudinal furrowed tongue	3.40	1.12	86.7% (13/15)	3.20	0.94	86.7% (13/15)	Yes
6 Cold peripheries	3.00	0.85	80.0% (12/15)	3.07	0.96	80.0% (12/15)	Yes
7 Dry incontinence pad	3.67	1.05	93.3% (14/15)	3.67	1.11	93.3% (14/15)	Yes
8 Change in urine color	4.00	1.31	80.0% (12/15)	4.07	1.34	80.0% (12/15)	Yes
9 Change in behavior	4.20	0.78	100.0% (15/15)	4.13	0.74	100.0% (15/15)	Yes
10 Reluctance to drink water*	4.18 (*N* = 11)	1.25	90.9% (10/11)	4.30 (*N* = 10)	0.68	100% (10/10)	Yes*
11 Dry skin and skin turgor*	3.55 (*N* = 11)	1.37	72.7% (8/11)	3.80 (*N* = 10)	1.14	80% (8/10)	Yes*
12 Constipation*	3.64 (*N* = 11)	1.21	72.7% (8/11)	3.40 (*N* = 10)	1.17	70% (7/10)	No*
**Exemplary panelist quotes extracted verbatim**							
*Hydration is extremely important for older adults as usually they do not drink enough water and hydrating liquids*.							
*Refer to studies published by Bunn and Hooper at University of East Anglia which clearly demonstrate that all commonly used signs and symptoms of dehydration (including assessment of fluid intake, urine color, urine volume, dry mouth and feeling thirsty) lack even basic levels of diagnostic accuracy for dehydration, are not appropriate to use, and should not be relied on individually as ways of assessing either presence or absence of dehydration in older people*.							
**Expert panelist feedback: suggested revisions**							
New components/domain were suggested and included:							
‒ Reluctance to drink water ‒ Dry skin ‒ Skin turgor ‒ Constipation ‒ Edema evaluation							
**New domain 5 edema** *	4.09 (*N* = 11)	0.94	100% (11/11)	3.80 (*N* = 10)	1.32	80% (8/10)	Yes*
**Expert panelist feedback: no suggested revisions**							
**Domain 4 oral health**	4.60	0.63	100.0% (15/15)	4.53	0.64	100.0% (15/15)	Yes
**Components domain 4 oral health**							
1 Revised oral assessment guide or oral health assessment tool	4.40	0.63	100.0% (15/15)	4.20	0.68	100.0% (15/15)	Yes
2 Taste and smell	4.27	0.88	93.3% (14/15)	4.20	0.86	93.3% (14/15)	Yes
3 Swallowing function/dysphagia screening*	4.91	0.30	100% (11/11)	4.91	0.30	100% (11/11)	Yes*
**Exemplary panelist quotes extracted verbatim**							
*Oral health is sometimes neglected by health professionals on screening for malnutrition in older adults but it's extremely important*.							
*Adequate oral health is critical for consuming an adequate diet for all nutrients especially animal proteins. Older adults may hide that they have difficulty chewing and although there are less older adults with dentures today, many older adults have poor oral health due to dry mouth, missing teeth, cavities, gum disease, etc*.							
**Expert panelist feedback: suggested revisions**							
Two panelists recommended to include swallowing and dysphagia screening as a new component.							
**Three age categories** * “Young‐old” “Old‐old” “Oldest‐old”	4.09	1.22	81.8% (9/11)	4.00	1.18	81.8% (9/11)	Yes*
**Exemplary panelist quotes extracted verbatim**							
*Frailty rather than age*.							
*May be more useful in old‐old and oldest‐old. In young‐old there is a need to focus more on prevention*.							
*Clinical conditions amongst all age groups will stabilize for differences in ages*.							
**Expert panelist feedback: suggested revisions**							
The comments acknowledged the complexity of aging and advocate for individualized assessments that consider both age and health status							

*Note:* 5‐point Likert scale ranged from ‘strongly disagree’ (1), ‘somewhat disagree’ (2), ‘neither disagree nor agree’ (3), ‘somewhat agree’ (4), to ‘strongly agree’ (5).

^a^ Usefulness: Possible to be applied in clinical practice.

^b^ Relevance: Appropriate and significant in clinical practice.

* Component/Domain reached consensus in second Delphi round—Results shown from second Delphi round.

The open comments did not suggest modifying or removing any of the suggested domains and components. However, the experts suggested a new *Edema* domain and four new components.

Three of the four newly suggested components were for the domain *Dehydration*, and they included *Reluctance to drink water, Dry skin and skin turgor*, and *Constipation*. The other new component was *Swallowing function/Dysphagia screening* for the domain *Oral Health*. The four newly suggested components and the new domain *Edema* were reviewed employing a secondary literature review and confirmed as eligible for inclusion in the Delphi survey round two.

Besides the panelists' proposed new components and domains, many comments emphasized the importance of the already included domains and components. Apart from this, most open comments focused on the practicality, availability, and financing of specific measurement methods. Additionally, the comments stressed the importance of always putting these components into the context of the holistic nutritional assessment, particularly in the domain *Dehydration*. All domains, components, and the identified themes from the open‐ended question were returned to the experts anonymously for in‐round feedback. However, none of the panelists revised their ratings.

In round two, components that did not reach consensus in round one were included, as well as the newly proposed components and domain. Additionally, the question on the usefulness and relevance for all three age categories was presented again. Eleven out of the 15 initial experts participated in round two (response rate of 73.3%). If participants only completed one round of the Delphi survey, the results from round one were retained. One panelist answered only the question on the usefulness of components in the domains *Functional Status/Body Composition*, *Dehydration*, and *Oral Health*, but not the relevance. Otherwise, all questions were fully answered by all panelists. Consensus was reached in the second round for all remaining and newly proposed components except for *Constipation* in the domain *Dehydration* (Table 3). There were no suggestions for modifications, deletions, or new components, and there were fewer open comments in round two.

After two rounds, all but one component reached the a priori defined consensus. Therefore, the Delphi study was terminated after two survey rounds.

### 3.4 Domain 1—General

The domain *General* reached 100% consensus in round one, and the corresponding component *Clinical Frailty Score* also reached consensus in round one, with 93.3% of experts rating the Clinical Frailty Score as both useful and relevant (Table 3). No revisions were suggested for this domain, and the open comments stressed the importance of considering overall health status and frailty when assessing nutritional status in older adults.

### 3.5 Domain 2—Functional Status/Body Composition

The domain *Functional Status/Body Composition* was rated as both useful and relevant by 93.3% in the first round (Table 3). The domain included the three subdomains *Capacity in own social and physical context*, *Physical performance and muscle strength*, and *Muscle mass/Body composition*. The two subdomains, *Capacity in own social and physical context* and *Physical performance and muscle strength*, were both rated as both useful and relevant by 93.3%. All components of these two subdomains also reached consensus with at least 80% agreement (Table 3).

The subdomain *Muscle mass/Body Composition* would have reached consensus in the first Delphi round, with 93.3% rating the subdomain as useful and 86.7% as relevant (data not shown). However, several components of this subdomain did not reach consensus in the first Delphi round, so this subdomain and the components that did not reach consensus were presented to the panelists again in round two with revised instructions. The comments for this subdomain highlighted the need for pragmatic and accessible tools for nutritional assessment outside acute hospital settings, acknowledging the challenges associated with obtaining and interpreting advanced imaging methods due to cost, radiation exposure, and time constraints. The experts emphasized the preference for simpler and more readily available measurements, such as assessment of Activities of Daily Living, handgrip strength, and mid‐upper arm circumference in clinical practice, particularly in long‐term care settings where practicality and feasibility are crucial considerations. They also recognized that different tools or tests are needed in different clinical contexts. However, the panelists suggested no edits for this subdomain after round one. In round two of the Delphi survey, this subdomain reached 100% consensus, and all remaining components reached consensus.

### 3.6 Domain 3—Dehydration

The domain *Dehydration* reached 100% consensus for both usefulness and relevance in round one (Table 3). Additionally, all components of this domain reached consensus with at least 80% agreement. The open comments emphasized the critical importance of assessing hydration status as a core component in evaluating the nutritional status of older adults. Additionally, the comments stressed the importance of putting these components into the context of the holistic nutritional assessment, such as medication use, urinary tract infections, cognitive impairment, and physical limitations in fluid intake. The experts highlighted the complexity of assessing hydration status in older adults and emphasized using a combination instead of only single clinical signs to screen for dehydration. Furthermore, new components were suggested, including *Reluctance to drink water*, *Dry skin/skin turgor*, and *Constipation*. Based on a secondary literature search, the new suggested components were supported and thus added to round two of the modified Delphi survey. The newly suggested components, *Reluctance to drink water* and *Dry skin/skin turgor*, both reached consensus in round two, whereas the component *Constipation* did not reach consensus in round two.

### 3.7 New Domain—Edema

In round one, *Edema* evaluation was suggested as an additional component. After a secondary literature review, it was included in the Delphi Survey round two as a new domain. In round two of the Delphi survey, 100% of the panelists rated this domain as useful and 80% as relevant (Table 3). The panelists suggested no further revisions.

### 3.8 Domain 4—Oral Health

The domain *Oral Health* reached 100% consensus in round one (Table 3). The corresponding components, *Revised Oral Assessment Guide or Oral Health Assessment Tool*, and *Taste and Smell*, also both reached consensus with 100% and 93.3%, respectively. The open comments underscored the importance of assessing oral health as a critical aspect of nutritional evaluation in older adults and that oral health is sometimes neglected. *Swallowing function/Dysphagia screening* was suggested by two experts as a new component under this domain and included in round two after a secondary literature review. In round two, this new component, *Swallowing function/Dysphagia screening*, reached 100% consensus.

### 3.9 Three Age Categories

The classification into three age categories did not reach consensus in round one (data not shown). In the open comments, the experts acknowledged the complexity of aging and advocated for individualized assessments that consider both age and health/functional status. The comments highlighted the importance of focusing on prevention in young‐old individuals while recognizing the relevance of all domains, particularly in the oldest‐old age category, where changes are more common. The question was presented again in round two and reached 81.8% consensus in round two.

## 4 Discussion

We aimed to generate consensus on best practices for NFPE+ components specific to older adults using a modified Delphi approach with leading nutrition professionals in gerontology across the globe. A comprehensive literature review identified 30 potential NFPE+ components specific to older adults across the NCP assessment domains. Subsequently, all 30 proposed NFPE+ components and three newly suggested components achieved consensus through a structured Delphi survey with international experts in geriatric nutrition. These 33 components that met consensus were categorized into five NFPE+ domains in a finalized NFPE+ tool specific to older adults (Table 4).

**Table 4:** NFPE+ components and domains specific to older adults that achieved consensus—summary table.

General	Functional status/body composition	Dehydration	Edema	Oral health
Clinical Frailty Scale	Capacity in own social and physical context	Vital signs Rapid weight loss Capillary refill Dry oral mucosa Longitudinal furrowed tongue Cold peripheries Dry incontinence pad Change in urine color Change in behavior Reluctance to drink water Dry skin and skin turgor		Revised Oral Assessment Guide or Oral Health Assessment Tool Taste and smell Swallowing function/Dysphagia screening
	Activities of daily living Instrumental activities of daily living			
	Physical performance and muscle strength			
	Handgrip strength Chair stand test Balance test Walk test (4–400 m) Short Physical Performance Battery test Time Up and Go test Gait speed			
	Muscle mass/Body Composition			
	Dual‐energy X‐ray absorptiometry Computed tomography Magnetic resonance imaging Bio‐electrical impedance analysis Ultrasound Body mass index Mid‐upper arm circumference Calf circumference Waist circumference			

There have been few Delphi studies on nutritional assessment or NFPE specific to distinct populations. However, the characteristics of this Delphi study are similar to two recent works in which NFPE‐specific components were identified using the Delphi technique, one for sports nutrition and another for infants and children with bronchopulmonary dysplasia (BPD) [51, 52]. Bathgate et al. aimed to identify and obtain consensus on NFPE components specific to infants and children with BPD [51]. They recruited 19 RDNs through pediatric nutrition professional associations and conducted a two‐round Delphi process using an online survey [51]. Thirty‐eight components categorized into six domains achieved a priori defined 75% agreement for usefulness or relevance [51]. Similarly, Pike et al. conducted a two‐round Delphi process with RDN experts in sports nutrition to obtain consensus on previously identified NFPE components specific for collegiate, elite, and tactical athletes [52]. Five NFPE domains with 22 components specific to this population achieved a priori defined consensus [52]. This current research aimed to pursue a similar approach by determining NFPE+ specific for older adults across different NCP assessment domains, including but not limited to the traditional NFPE from the NCP [18]. While in the Bathgate et al. and the Pike et al. studies, all panelists were US RDNs [51, 52], this study recruited international experts in geriatric nutrition from eight different countries. Similarly to Bathgate et al. and Pike et al., [51, 52] only two Delphi rounds were needed to reach consensus, and all domains and components except for one newly suggested component by the panelists (*Constipation* as an indicator of dehydration) reached consensus.

Compared to the study by Bathgate et al., [51] the level of participation was higher in this modified Delphi study. In the study by Bathgate et al., [51] the questions at the beginning of the survey were answered by more panelists, and the questions that appeared later in the survey had a decreasing response rate; in this study, all panelists had completed all rating questions on the 5‐point Likert scale in the first round. Similarly to Bathgate et al., [51] consensus was reached in the first round for all four proposed domains and 24 of the 30 proposed components. All six components that did not reach consensus were from the domain *Functional Status/Body Composition* in the subdomain *Muscle mass/Body Composition*. Panelists' open comments indicated that not only the usefulness and relevance of a physical domain or component but also the practicality, availability, and financial considerations were rated. Additionally, the panelists' ratings seemed to reflect what is currently being done in clinical practice. Therefore, the instructions for the experts were updated for the second Delphi survey round. The revised instructions emphasized that the experts should consider what would be useful and relevant if available to the clinician and also in different practice settings, such as inpatient, outpatient, private practice, and so forth. In the second Delphi survey round, all six components from the domain *Functional Status/Body Composition* in the subdomain *Muscle mass/Body Composition* reached consensus with a higher percentage of agreements and a higher mean for both usefulness and relevance.

While all proposed domains and components reached consensus, one newly suggested component by the panelists, *Constipation* (as an indicator of dehydration), did not reach consensus. Constipation is highly prevalent in older adults, with studies showing that 15%–30% of older adults have constipation [69, 70]. Inadequate fluid intake is considered a common cause of constipation [71]. However, the current evidence regarding the association of hydration status and constipation is weak [71]. Furthermore, besides nutritional and hydration status, inactivity, weakened abdominal and pelvic floor muscles combined with age‐related changes in bowel function, polypharmacy, and chronic illness are among the most important risk factors for constipation in older adults [70]. Due to the multifactorial causes of constipation, experts might not have considered constipation as a useful and relevant physical sign of dehydration [71].

Overall, and particularly for the domain *Dehydration*, the open comments emphasized the importance of always using a combination of clinical signs (as opposed to single tests/measurements) to screen for dehydration in older adults. Up to 25% of nonhospitalized and up to 60% of community‐dwelling older adults show signs of dehydration [6, 9, 72, 73, 74]. Older adults are at high risk for dehydration due to multifactorial causes that include but are not limited to age‐related declines in thirst sensation and kidney function, memory loss, dysphagia, physical limitations (e.g., difficulty to twist open a water bottle, having the strength to walk to the kitchen and get a drink), or social isolation [6, 9, 75, 76, 77, 78, 79, 80, 81]. The ESPEN guideline recommends against physical signs and instead highlights direct measurement of serum/plasma osmolality as the gold standard for dehydration detection in older adults [9]. For example, skin turgor might not be reliable due to the loss of subcutaneous tissue of the aging skin [6]. However, other publications recommend using a combination of clinical signs to screen for dehydration in older adults, particularly in the absence of laboratory test availability [6, 23]. These recommended physical signs were included in this Delphi study and comprised dry incontinence pad due to decreased urine output, change in urine color, change in behavior, lower blood pressure (< 100 mmHg), and higher pulse (> 90 beats/min.), increased respiratory rate (> 20 breaths/min) dry mucosa and longitudinal furrowed tongue, rapid weight loss (> 1 kg/d), and increased capillary refill time (> 2 s) or cold peripheries [6, 23]. These physical signs and tests are vital for assessing dehydration in nursing home residents or older adults in other settings where relevant diagnostic methods are not feasible [6]. Initially and regularly evaluating NFPE+ components can contribute to avoiding unnecessary and time‐consuming invasive tests and prevent avoidable hospital admissions [6].

In future studies, the two domains of *Edema* and *Dehydration* could be combined as *Hydration*/*Fluid Status*. One reason this was not already done in this study is that the initial literature search showed that even though edema is a common health problem in older adults, the components of the NFPE for assessing edema can be applied to older individuals in the same way as to younger adults. In comparison, different tests and assessments are recommended for dehydration in older adults than in younger adults. Therefore, this Delphi study focused on dehydration signs and tests in the first Delphi survey round and not overall fluid status.

To capture the experts' opinion on the differences between the various age groups among older adults (‘young‐old’, ‘old‐old’, and ‘oldest‐old’ individuals) [12, 13], the experts were also asked in round one whether all the identified domains and components were useful and relevant for all three age categories. This question did not reach consensus in round one and was commented on by many panelists. The comments stressed the importance of considering the complexity of aging and advocated for individualized and critical assessments that account for age, geographical location (e.g., to consider First Nations people), and medical status or frailty score. Therefore, all NFPE+ domains and components are useful and relevant for all age groups. However, the NFPE+ components do not need to be evaluated at every nutrition assessment; they should be assessed only if clinically indicated.

### 4.1 Limitations and Strengths

This modified Delphi study had several limitations, especially concerning the Delphi technique [46]. First, the pre‐selection of NFPE+ domains and components may be personally biased by the authors. However, they were confirmed by the comprehensive literature search with documented references. Second, there is no clear indication of an appropriate number of experts for the Delphi panel or clear inclusion criteria [46]. Therefore, there is a risk that an inadequate number or insufficient representative expert panel was recruited. The same applies to the definition of consensus, where there is no clear indication of the minimum percentage of consensus required [46]. Additionally, in this study, the 75% consensus included ‘neither disagree nor agree’ and not only ‘somewhat agree’ and ‘strongly agree’. Last, the entire Delphi process and each Delphi round were time‐consuming. The experts were not compensated for their participation, and many surveys are currently being conducted in the dietetic community. This could have led to survey fatigue, corresponding recruitment difficulties, or a higher drop‐out rate.

One strength of this study was the international makeup of the Delphi panel. Despite phenotypic differences (e.g., American vs. European older adults), consensus was achieved for 33 NFPE+ components, and the panelists' open‐ended comments were similar despite these potential phenotypic differences. Another strength was the study design, whereby the survey was based on a comprehensive literature search with the initial identification of appropriate NFPE+ components for older adults.

### 4.2 Implications

In clinical practice, it is not the aim that all NFPE+ domains and components must be evaluated at every nutritional assessment, but only if clinically indicated. The nutritional assessment and the corresponding NFPE+ components identified in this Delphi study may also differ depending on the setting (e.g., hospital vs. nursing home), since different diagnostic options may be available depending on the setting. For example, in a hospital, serum/plasma osmolality can be measured as the gold standard for dehydration detection in older adults, but in a nursing home, clinicians may need to rely on the identified NFPE+ components. Furthermore, the dietitian is not expected to directly measure or obtain information regarding all physical domains or components (e.g., Chair stand test, vital signs, MRI). Physical findings (abnormal or normal) can also be obtained through other healthcare professionals or medical records. For example, if a doctor performs a geriatric assessment, the dietitian may not collect the existing NFPE+ components again but should include the results in their assessment.

The 33 NFPE+ components and five NFPE+ domains that achieved consensus will be combined as a finalized NFPE+ tool specific to older adults. This finalized NFPE+ tool could be tested in future validation and reliability studies. The ultimate goal is a tool that can be implemented internationally in the field. In this context, one future research question could include which domains and components are significant for which age categories or whether medical status or frailty score is more important than age when assessing the relevant assessment components. Another future research question could include the scope for nutritional assessment variation depending on the level of dietetic competency (e.g., competent vs. proficient vs. expert).

Education and training for the incorporation into geriatric nutrition care will be needed as soon as the finalized NFPE+ tool specific to older adults has been validated. While NFPE has long been recognized and implemented as an essential part of the nutritional assessment in the United States [82], this may not yet be the case in other countries. It is, therefore, possible that training needs may differ internationally.

### 4.3 Conclusion

Older adults are a growing population, often suffering from multiple chronic diseases and nutrition‐related problems. Therefore, NFPE+ domains and components specific to older adults may significantly contribute to a comprehensive nutritional assessment in older adults. Fifteen experts in geriatric nutritional care from across the globe participated in this modified Delphi survey and reached consensus regarding the most relevant and useful NFPE+ components for older adults. The final 33 components were categorized into the five NFPE+ domains: *General*, *Functional Status/Body Composition*, *Dehydration*, *Edema*, and *Oral Health*. This final NFPE+ tool specific to older adults could be tested in future validation and reliability studies with the ultimate goal of a tool that can be implemented internationally in the field.

## Author Contributions

**Christina E. Gassmann:** conceptualization, methodology, formal analysis, data curation, writing, review, and editing. **Diane L. Rigassio Radler, Laura Byham‐Gray, Caroline M. Kiss** and **Alainn Bailey:** conceptualization, methodology, review, and editing.

## Conflicts of Interest

The authors declare no conflicts of interest.

## 1 Peer Review

The peer review history for this article is available at https://www.webofscience.com/api/gateway/wos/peer-review/10.1111/jhn.70101.

## 2 Transparency Declaration

The lead author affirms that this manuscript is an honest, accurate, and transparent account of the study being reported. The lead author affirms that no important aspects of the study have been omitted and that any discrepancies from the study as planned (Institutional Review Board of Rutgers University, New Jersey, United States of America, Pro2024000274) have been explained.

## Data Availability

The data that support the findings of this study are available on request from the corresponding author. The data are not publicly available due to privacy or ethical restrictions.
